# Broad-Spectrum Adverse Events of Special Interests Based on Immune Response Following COVID-19 Vaccination: A Large-Scale Population-Based Cohort Study

**DOI:** 10.3390/jcm14051767

**Published:** 2025-03-06

**Authors:** Hong Jin Kim, Jee Hyun Suh, Min-Ho Kim, Myeong Geun Choi, Eun Mi Chun

**Affiliations:** 1 Department of Orthopaedic Surgery, Kyung-in Regional Military Manpower Administration, Suwon 16440, Republic of Korea; hongjin0925@naver.com; 2 Department of Orthopaedic Surgery, Inje University Sanggye Paik Hospital, College of Medicine, Inje University, Seoul 01757, Republic of Korea; 3 Department of Rehabilitation Medicine, College of Medicine, Ewha Womans University, Seoul 07985, Republic of Korea; jeehyun.suh1@gmail.com; 4 Informatization Department, Ewha Womans University Seoul Hospital, Seoul 07804, Republic of Korea; mino-kim@naver.com; 5 Department of Internal Medicine, Division of Pulmonology and Critical Care Medicine, School of Medicine, Ewha Womans University, Seoul 07985, Republic of Korea; cmk1006@nate.com

**Keywords:** COVID-19, vaccination, adverse events of special interests, cumulative incidences, risks

## Abstract

**Background/Objectives:** Current studies on adverse events related to the COVID-19 vaccine have predominantly focused on severe, life-threatening side effects. However, numerous less severe but common adverse events (AEs) remain underreported and insufficiently investigated despite their potential impact. **Methods:** This population-based cohort study investigated the cumulative incidence rate (cIR) and risk of the broad-spectrum AEs of special interests (AESIs) based on immune response, including gynecological, dermatological, ophthalmological, otologic, and dental problems, following COVID-19 vaccination. **Results:** Among 4,203,887 individuals in Seoul, South Korea, the final analysis included 1,458,557 vaccinated subjects and 289,579 non-vaccinated subjects after the exclusion of underlying diseases. The cIR of AESIs for three months was significantly higher in vaccinated subjects than in non-vaccinated subjects, except for endometriosis. The vaccination significantly increased the risks of all the AESIs except for visual impairment. The risk of alopecia showed the highest HRs (HR [95% CI] = 2.40 [1.90–3.03]) among the AESIs following COVID-19 vaccination. Among the vaccinated subjects, heterologous vaccination was associated with the increased risk of most of the AESIs. **Conclusions:** Our findings suggest that clinicians should closely recognize and follow up on various COVID-19 vaccine-related AEs due to their unknown impact, even if they may not be serious at present.

## 1. Introduction

Coronavirus disease-2019 (COVID-19), as a global challenge for health and socioeconomic issues, demonstrated a subsequent rise in morbidity and mortality in the early stages of the pandemic compared to other viral infections [1]. With the rapid development of vaccines in response to the unprecedented COVID-19 pandemic, there has been a contribution to the reduction of the severity and fatality rates. Subsequently, several types of vaccines after approval of the AZD1222 vaccine have been released to prevent COVID-19 infection [2,3,4,5]. However, a wide range of adverse events (AEs) of special interests (AESIs), not previously reported in conventional vaccines, have been observed post-vaccination [6,7,8,9]. Lee et al. investigated AEs associated with COVID-19 vaccines between 28 February and 21 August 2021 [10]. In this study, the incidences of AEs caused by COVID-19 vaccination varied based on age, sex, and dose order [10]. In particular, various AEs were shown in all types of COVID-19 vaccines [10].

As every country has performed, South Korea initiated the COVID-19 vaccination program at care facilities and subsequently expanded its coverage to encompass the entire nation’s population. About 80% of the population in South Korea was vaccinated within a year, which contributed to a significant decrease in COVID-19 infection [9]. Meanwhile, there is increasing evidence that many vaccinated populations could experience several unexpected complications, such as AEs, based on the immune responses [6,7,8,10,11]. Many post-vaccination AEs are believed to originate from an immune response characterized by an inflammatory cytokine storm that causes irreversible damage to the cardiovascular, cerebrovascular, and respiratory systems [12,13].

Much of the current literature on AEs following COVID-19 vaccination has focused on serious AEs, such as cardiovascular complications, which limit daily activities by more than 50%, necessitate hospitalization, or pose life-threatening risks [10]. Given this background, our particular interest lies in non-serious but common AEs, which have yet to be comprehensively reported [11,14,15,16,17,18,19,20]. Based on the immune-based inflammatory reactions following COVID-19 vaccination, we investigated 13 broad-spectrum AEs that are potentially linked to the immune-mediated mechanisms of COVID-19 vaccination. Therefore, this study aims to assess the AEs after COVID-19 vaccination from the National Health Insurance Service (NHIS) database in Seoul, South Korea.

## 2. Materials and Methods

### 2.1. Data Source

This from the Korean NHIS database on 1 January 2021 enrolled randomly extracted 50% of individuals residing in Seoul, South Korea, as a representative sample. We randomly selected 50% of the residents living in Seoul as of 1 January 2021. The International Classification of Diseases, 10th revision (ICD-10), was adopted by the NHIS to classify disease diagnoses. The data included the primary diagnosis, secondary diagnosis, and dates of hospital visits. This population-based cohort study was conducted using the Strengthening the Reporting of Observational Studies in Epidemiology (STROBE) guidelines [21].

### 2.2. Study Population

A total of 4,348,412 individuals living in Seoul, constituting 50% of the population, were included and investigated as of 1 January 2021. Individuals aged under 20 years were excluded, leaving 4,203,887 individuals for analysis. In this study, only individuals who had received two doses of COVID-19 vaccine were included in the vaccinated group. In this cohort study, the index date, which is the date on which individuals started participating in the study, was set differently for the vaccinated and non-vaccinated groups. For the vaccinated group, the index date was set as the date of the second vaccine dose administered before 1 October 2021. On the other hand, for the non-vaccinated group, the index date was set as 1 October 2021 to ensure at least a 3-month observation period. The vaccinated group included 3,839,014 individuals, whereas the non-vaccinated group included 364,873 individuals. Individuals who received a dose of vaccine before 1 January 2021 and did not receive a second dose between 1 January 2021 and 1 October 2021 were excluded. The vaccinated group included 2,154,389 individuals, whereas the non-vaccinated group included 350,953 individuals.

The broad-spectrum AEs that are non-serious but common include gynecological (endometriosis [N80], and menstrual disorders [N92; polymenorrhagia, menorrhagia, abnormal cycle length, oligomenorrhea, and amenorrhea]), hematological (bruises [D69] confined to non-tender and yellow-colored on especially extremities), dermatological (herpes zoster [B02], alopecia [L63], and warts [B07]), ophthalmological (visual impairment [H54], and glaucoma [H40, H42]), otological (tinnitus [H93.1], inner ear [H81-83], middle ear [H65-67], and outer ear [H90-94] disease), and dental problems (periodontal disease [K05]) as reported by the Vaccine Adverse Event Reporting Center. The diagnostic records for a year before the index date were traced. Individuals with any target disease as a primary or secondary diagnosis during this period were excluded from the study. The occurrence of the target disease was defined as receiving a primary diagnosis of the disease from the day after the index date.

### 2.3. Outcome Measurements

The primary outcome measure was cumulative incidence rates (cIRs) of AEs per 10,000 population between the vaccinated and non-vaccinated subjects. The cIRs of the AEs were measured at one week, two weeks, one month, and three months. To monitor the occurrence of target AEs up to three months post-vaccination, we tracked different time points: one week and two weeks to assess immediate and acute responses, one month to capture delayed-onset events, and three months as the final follow-up in this study. The secondary outcome measures were the odd ratios (ORs) and hazard ratios (HRs) of AEs. Furthermore, subgroup analyses were also conducted based on gender, the number of COVID-19 vaccine doses, the vaccine type (mRNA vaccine [Vaccination using only mRNA-based vaccine platforms], cDNA vaccine [Vaccination using only cDNA-based vaccine platforms], and heterologous vaccination [When the first and second doses were administered using different vaccine platforms]), health insurance level, presence of diabetes mellitus (DM), hypertension (HTN), hyperlipidemia, and chronic obstructive pulmonary disease (COPD).

Age, gender, insurance level, Charlson’s comorbidity index (CCI), presence of DM, HTN, hyperlipidemia, and COPD, and prior COVID-19 infection history were extracted using their ICD-10 codes, which were suggested by Sundararajan et al. The presence of comorbid diseases (i.e., DM, HTN, hyperlipidemia, and COPD), items in CCI, and the prior COVID-19 infection history was determined as a primary or secondary diagnosis 2 or more times within 1 year before the index date [22]. The NHI premium was used as a proxy measure of income because it is proportional to monthly income, including earnings and capital gains. The income quantiles of the enrolled individuals were categorized into three groups (low-, middle- and high-income groups in medical aid enrollees and the 0–33, 34–66, and 67–100 centiles of NHI enrollees).

### 2.4. Statistical Analysis

Statistical analysis was performed using the SAS Enterprise Guide (version 8.3., SAS Institute, Cary, NC, USA). The normality of data distribution was assessed using the Kolmogorov–Smirnov test. Baseline patient characteristics and comorbidities were reported as means ± standard deviation for continuous variables and as frequencies (percentages, %) for categorical variables. For group comparisons, Student’s *t*-test was used for continuous variables, while categorical variables were analyzed using the chi-square test or Fisher’s exact test when appropriate. Immune-mediated adverse events associated with COVID-19 vaccination were also assessed using these statistical methods. The cIR was calculated as the occurrence of events per 10,000 individuals. To evaluate the association between COVID-19 vaccination and adverse events, we employed a multiple logistic regression model to estimate odds ratios (ORs) with corresponding 95% confidence intervals (CIs). Additionally, Cox proportional hazards regression was used to estimate hazard ratios (HRs) with 95% CIs. For both multiple logistic regression and Cox proportional hazards regression models, comorbidities were included as covariates to adjust for potential confounding factors. A two-sided *p*-value of ≤0.05 was considered statistically significant.

## 3. Results

### 3.1. The Participants’ Characteristics

**Figure 1:**
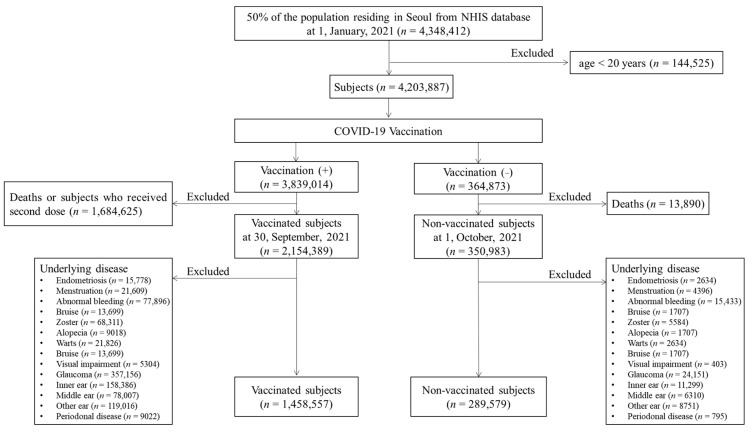
Flowchart of this study.

In total, 1,748,136 subjects were included in this study. Among them, 289,579 (16.57%) had not received the COVID-19 vaccine (i.e., non-vaccinated subjects), whereas 1,458,557 (83.43%) were vaccinated against COVID-19 (i.e., vaccinated subjects) (Figure 1). The baseline characteristics of the vaccinated and non-vaccinated groups are shown in Table 1.

### 3.2. The cIRs per 10,000 of the Non-Serious AEs Following the COVID-19 Vaccination

Among the broad-spectrum AEs in this study, the cIRs at three months following COVID-19 vaccination were higher in vaccinated subjects than in non-vaccinated subjects, except for endometriosis. The highest cIR of the broad-spectrum AESIs in vaccinated subjects was observed in other ear diseases (cIR, 51.78; 95% CI, 50.61–52.94) followed by inner ear diseases (cIR, 47.10; 95% CI, 45.99–48.21), herpes zoster (cIR, 45.08; 95% CI, 43.99–46.17), menstrual disorders (cIR, 44.43; 95% CI, 43.35–45.51), and glaucoma (cIR, 39.42; 95% CI, 38.40–40.43). Among the broad-spectrum AESIs, 50% exhibited a significant difference in cIRs one week post-vaccination. Menstrual disorders and visual impairments were noted for one month, whereas alopecia, warts, and periodontal disease were observed from two weeks onwards (Table 2).

When stratified by gender, the cIRs of broad-spectrum AESIs showed a similar pattern to that of the overall population. At three months post-vaccination, menstrual disorders presented the highest cIRs in females (cIR, 87.54; 95% CI, 85.41–89.66) followed by inner ear diseases in females (cIR, 62.82; 95% CI, 61.02–64.62), other ear diseases in females (cIR, 55.27; 95% CI, 53.58–56.96), and herpes zoster in females (cIR, 53.13; 95% CI, 51.48–54.79) (Table S1). When stratified by vaccine type, heterologous vaccination increased the cIR of menstrual disorders to 78.96 (95% CI, 73.45–84.48) (Table S2). Detailed further information on AE risk based on gender is presented in Table S3.

### 3.3. The Risks of Non-Serious AEs Following the COVID-19 Vaccination

**Figure 2:**
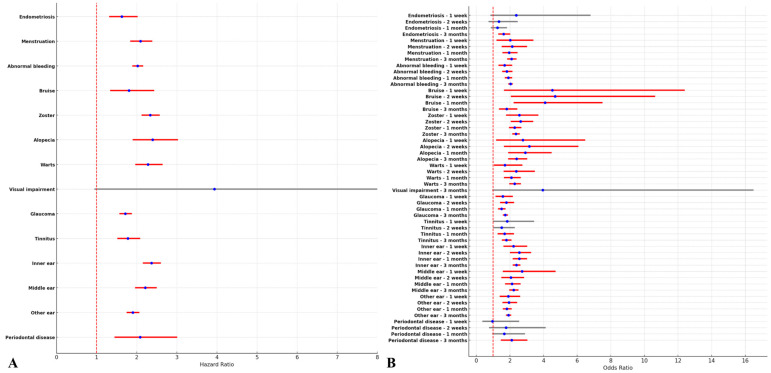
The risk for the broad-spectrum adverse events of special interests (AESIs) following COVID-19 vaccination. (**A**) Cox proportional hazard model, which is presented as a forest plot (hazard ratio [red circle] with 95% confidence interval [bar]). If clinical significance, it is marked as red-bar. (**B**) The multivariate logistic regression model, along with the time point, is presented as a forest plot (odd ratio [red circle] with 95% confidence interval [bar]).

In the Cox proportional hazard model in this study, COVID-19 vaccination significantly increased the risks of the broad-spectrum AESIs except for visual impairments (HR, 3.94; 95% CI, 0.94–16.41), with the highest level of alopecia (HR, 2.40; 95% CI, 1.90–3.03) followed by inner ear diseases (HR, 2.37; 95% CI, 2.15–2.60), and herpes zoster (HR, 2.34; 95% CI, 2.12–2.57) (Figure 2A). In the multivariate logistic model in this study, the COVID-19 vaccination was associated with a significant increase in the risk of most broad-spectrum AESIs, indicating potential influence in the early time point (one week after COVID-19 vaccination). At three months post-vaccination, the COVID-19 vaccination significantly increased the risk of endometriosis (OR, 1.63; 95% CI, 1.31–2.03). Furthermore, visual impairment at three months (OR, 3.96; 95% CI, 0.95–16.50), tinnitus at one week (OR, 1.84; 95% CI, 0.98–3.45), and two weeks (OR, 1.51; 95% CI, 1.00–2.29), and periodontal diseases at one-week (OR, 0.97; 95% CI, 0.37–2.55), two weeks (OR, 1.77; 95% CI, 0.76–4.13), and one-month (OR, 1.66; 95% CI, 0.96–3.04) showed no statistical differences of ORs between two groups (Figure 2B).

### 3.4. The Risks of the Broad-Spectrum AESIs According to the COVID-19 Vaccine Type

**Figure 3:**
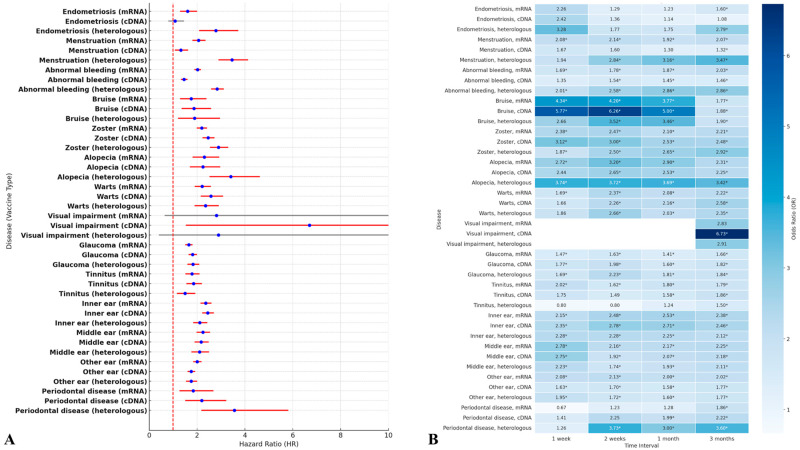
The risk for the broad-spectrum adverse events of special interests (AESIs) of vaccinated subjects according to vaccine type. (**A**) Cox proportional hazard model, which is presented as a forest plot (hazard ratio [red circle] with 95% confidence interval [bar]). If clinical significance, it is marked as a red bar. (**B**) The multivariate logistic regression model, along with the time point, was presented as the odd ratio of a heatmap. If clinical significance, it is marked as *.

Both the multivariate logistic regression model and the Cox proportional hazard model were used to assess the risk factors according to the COVID-19 vaccine type. In the Cox proportional hazard model, heterologous vaccination was more increased the risks of gynecological problems, including endometriosis (HR, 2.78; 95% CI, 2.08–3.72]) and menstrual disorders (HR, 2.84; 95% CI, 2.58–3.12]), hematological problem including bruise (HR, 1.89; 95% CI, 1.21–2.96), dermatological problems including herpes zoster (HR, 2.89; 95% CI, 2.15–2.60) and alopecia (HR, 3.41; 95% CI, 2.52–4.62), ophthalmological problem including glaucoma (HR, 1.83; 95% CI, 1.60–2.09), and periodontal diseases (HR, 3.56; 95% CI, 2.18–5.82) compared to other types of vaccination (Figure 3A). In the multivariate logistic regression model, most risk trends showed a similar pattern to that of Cox proportional hazard models. According to time points, alopecia and periodontal diseases were associated with the highest risk outcomes following heterologous vaccinations compared to other vaccination methods. However, vaccination using cDNA only was observed to notably increase risks of bruising at one week (OR [95% CI] = 5.77 [2.01–16.54]), two weeks (HR, 6.26; 95% CI, 2.28–14.62), and one-month (HR, 5.00; 95% CI, 2.66–9.39]) (Figure 3B).

## 4. Discussion

### 4.1. Research Findings and Clinical Implications

The development of COVID-19 vaccines has been crucial in overcoming the COVID-19 pandemic by reducing disease severity and mortality. In other words, while COVID-19 vaccines have made significant contributions to the benefits of public health globally, there have also been reports expressing concerns about their safety due to the unprecedented speed of their development. The concerns for post-sequelae and vaccine-related complications have been raised as one of the important global issues [1,9]. The main focus of the COVID-19 vaccine-related complications were serious AEs, such as cardiovascular and neurological problems that can give rise to fatal conditions [6,7]. To the best of our knowledge, the research regarding non-serious AEs following COVID-19 vaccination did not conduct a large population-based cohort study at the nationwide level [10]. Here, we investigated the 14 non-serious AEs following COVID-19 vaccination in Seoul, South Korea. By comparing the 1,458,557 vaccinated subjects and 289,579 non-vaccinated subjects, we found that the broad-spectrum AEs, except for endometriosis, showed a significant increase in cumulative incidence following COVID-19 vaccination with notable significant higher rates of menstrual disorders, herpes zoster, glaucoma, inner ear diseases, middle ear diseases, and other ear disease than in non-vaccinated subjects. Furthermore, bruises are associated with COVID-19 vaccination in the early phase, showing the highest ORs in one week and two weeks. For three months of follow-up, alopecia showed the highest level of HRs following the COVID-19 vaccination. Therefore, our findings indicate that non-serious AEs commonly occur during the acute post-vaccination period. However, even if the risk appears elevated, our study does not account for the severity of each condition, necessitating a cautious interpretation.

Lee et al. presented mild AEs following COVID-19 vaccination, showing skin-related symptoms (23.6%) [10]. These findings are consistent with our results on skin-related disorders such as bruise. Meanwhile, for mild AEs, females have higher incidences than males [10]. The broad-spectrum non-serious AEs in this study (Table S1) presented that females have higher cIs than males, supporting a previous study [10]. Furthermore, almost all AEs showed a significantly increased risk regardless of vaccine type, though the magnitude of risk varied by vaccine type, consistent with previous studies [10]. Notably, our study further analyzed patterns over different time periods, suggesting that the level of risk may change over time.

### 4.2. The Type of Vaccination with the Association of Immune Response

The type of vaccination has been associated with immune response given cellular mechanisms [13,23]. Lee et al. showed that heterologous vaccination leads to enriched B cells and CD4+ T cell responses with higher activation of interferon pathways, suggesting the potential increase of irAEs [24]. In this study for the broad-spectrum AEs, heterologous vaccination showed the highest risks of eight AEs, including endometriosis, menstrual disorders, bruises, herpes zoster, alopecia, glaucoma, and periodontal diseases, compared to other types of vaccination. Thus, peripheral blood and skin lesions may exhibit heightened immune responses following heterologous vaccination.

### 4.3. The Role of Spike Protein in COVID-19 Vaccines

The spike protein is considered a primary target for the development of vaccines against COVID-19 because the infection by severe acute respiratory syndrome coronavirus 2 (SARS-CoV-2) is initiated by the binding of spike protein to the ACE2 receptor on the host cell surface [23,25]. Yonker et al. suggested that the circulating spike protein was detected in the peripheral blood of patients who developed post-mRNA vaccine myocarditis [25]. Several studies supporting Yonker et al. provide potential insight into the possibility that mRNA-LNP can act as a potential underlying cause for diverse AEs [25,26,27,28,29,30,31,32]. The main difference between cDNA-based vaccines and mRNA-based vaccines against SARS-CoV-2 was mediators of immune responses, which were respectively spike protein and lipid-nanoparticle-encapsulated mRNA [23]. The current hypothesis between COVID-19 vaccination and ear diseases as AEs is that the ear disease is activated by intensification of a spike protein-specific IgG and potential systematic immune response, suggesting the immunologic important factors [29]. Surprisingly, in this study, most ear problems, including tinnitus, inner ear disease, and middle ear diseases, showed a high level of HRs in vaccination using cDNA only compared to other types of vaccination. The findings of this study are not only consistent with existing hypotheses indicating the important role of spike protein in SARS-CoV-2. However, since our findings are based on observational data, the causal relationship needs to be further elucidated in future studies.

### 4.4. Gynecological AEs

For gynecological issues, previous studies have suggested that the COVID-19 vaccination increased bleeding from the changes in the irregular menstrual cycle [30]. Population-based studies reported that unexpected vaginal bleeding and menstrual bleeding changes as an emerging phenomenon [27,28]. In particular, menstrual changes caused by COVID-19 vaccination may be potentially concerning for younger women, as they could have adverse effects on fertility. Menstruation and ovulation are caused by physiological inflammation, which is related to TNF-α for folliculogenesis, IL-6 and -8 for the secretion of ovarian hormones, and the regulatory T cells for the luteal phase [33]. The spike protein binds to angiotensin-converting enzyme-2 and plays a role in cytokine storm [34]. Therefore, these immune cell activation and cytokine storms can potentially dysregulate menstruation [33,34]. Likewise, our study also showed increased cIRs and risks of menstrual disorders (including menorrhagia, metrorrhagia, and hypermenorrhea), which significantly rose on heterologous vaccinations. Considering previous studies, these manifestations were caused by hormonal changes resulting from spike proteins and disruption of the coagulation pathway in the endometrium [27,28]. While there were no significant differences in cIR of endometriosis at three months between vaccinated and non-vaccinated subjects, this study revealed an increased risk of endometriosis associated with COVID-19 vaccination at the three months post-vaccination. These results suggest that the cIR of endometriosis may see a significant rise beyond three months. On the other hand, alopecia, despite a significant increase in risk, exhibited a gradual decline over time. This suggests that the temporal trend of risk may vary depending on the condition during long-term follow-up. Therefore, conducting long-term analyses to observe epidemiological trends will be crucial in future research.

### 4.5. Dermatological AEs

One of the important points for this result was the trend of the diminishing risk of bruises after vaccination. Furthermore, the COVID-19 vaccine-associated bruise presented non-tender yellow-colored bruises, especially on the extremities. Specifically, the risk of bruising is highest around two weeks after vaccination and then decreases. This pattern suggests that careful monitoring is necessary during the acute post-vaccination period. Thus, both clinicians and vaccinated subjects need to be cautious of bruise occurrence within one-month post-vaccination. In addition, vaccination using cDNA only significantly increased the risks of bruises at the early phase compared to other types, so special caution may be needed in the vaccinated subjects using the cDNA vaccine for at least two weeks post-vaccination.

Considering the risk of most dermatologic AEs significantly increased following COVID-19 vaccination, the possible mechanism is molecular mimicry caused by spike protein [17,35]. This phenomenon can potentially lead to an increased risk of autoimmune AEs, as reported in the literature [35]. In line with this, we believe that T-cell activation may be crucial for the occurrence of dermatological COVID-19 vaccine-related AEs [17]. Although our finding has a limitation as an observational study, suggested plausible biological mechanisms could be verified in the future.

### 4.6. Strengths in This Study

Our study has several strengths for broad-spectrum AEs following COVID-19 vaccination. First, the broad-spectrum AESIs have been reported as case reports or case series, so it is the first study to investigate broad-spectrum AEs that are non-serious but common following COVID-19 vaccination [13,14,15,20,29,31]. For diverse manifestations, including post-vaccination glaucoma, ear diseases, and alopecia, these AEs shared potential pathophysiological mechanisms as an expression of spike proteins, which are associated with dysregulation of immunity [13,15,32]. With the suggested mechanisms in the literature, our studies may be consistent with this hypothesis because the types of vaccinations have higher HRs in heterologous or cDNA vaccination compared to mRNA-only vaccination. However, our hypothesis regarding this association needs to be validated through future experimental studies. Second, the current studies have demonstrated that the COVID-19 vaccine affects T cell-mediated immune response in multiple sclerosis, which leads to autoimmunity [19]. Our findings for the broad-spectrum AEs strengthen their studies, sharing similar pathophysiological mechanisms and hypotheses, such as the role of spike proteins and autoimmune diseases triggered by vaccines. However, the causal relationship needs to be verified in the future. Last, the non-serious AEs after COVID-19 vaccination are relatively common and can be affected by various factors such as vaccination methods [19]. Furthermore, our findings also suggest that different types of vaccinations exhibit distinct activation patterns at various sites, which will need to be studied in the future. Even in this case, warts on the cheek were developed with positive for spike IgG and negative for nucleocapsid IgG after mRNA-based vaccination, suggesting that the mRNA-LNP triggers an autoimmune response [18] COVID-19 vaccines may not be fatal, but individuals with a predisposition could be more vulnerable to a wide range of AEs. Furthermore, our study indicates that it is essential to consider potential side effects that persist beyond three months. As the toxicity of COVID-19 diminishes and a significant portion of the worldwide population acquires natural immunity, it is important to designate as vaccine recipients those for whom the benefits of vaccination outweigh the potential side effects of ongoing vaccination.

### 4.7. Limitations in This Study

This study has several limitations. First, our study has statistical inequity between the two groups, which is caused by a general population-based study. One of the main purposes of this study is to report the cumulative incidence of broad-spectrum non-serious AEs in the population residing in Seoul, South Korea, as a preliminary analysis using crude data. However, for broad-spectrum AESIs that are non-serious but common, large-sized population studies were scarce, so studies through the process of propensity score matching may be necessary for future research as a new paper. Second, target AEs were extracted based on ICD-10 codes in the claim databases, so coding, mismatching, or misclassification errors could have occurred. Third, we tried to broad-spectrum non-serious AEs following COVID-19 vaccination, but there is a possibility that some diseases may not be included. Furthermore, our study did not provide sub-group analysis based on age, gender, and intervals between 1st and 2nd vaccination. Last, additional confounding factors were not considered in this study, such as autoimmune disease or social factors, which could potentially result in selection bias, including healthy vaccine bias. Long-term studies are needed on the duration of AEs and on inflammatory disease-related side effects that did not appear in the short-term duration, which are underway to elucidate the AEs of COVID-19 vaccination. These limitations in our study should be illustrated through new research by specialty in the future.

## 5. Conclusions

The three-month risks of incidental broad-spectrum non-serious AEs are substantially higher in the COVID-19 vaccinated subjects than in non-vaccinated controls. Our findings suggested that vaccinated subjects with predisposition are potentially vulnerable to the occurrence of diverse AEs, although the COVID-19 vaccines may not be fatal. Consequently, clinicians should maintain closed monitoring of the broad-spectrum AEs after vaccination, given that these manifestations might emerge post-vaccination.

## Figures and Tables

**Table 1:** Baseline characteristics of the patients stratified by COVID-19 vaccination in South Korea.

	Total (*n* = 1,748,133)	Vaccination		*p*
		No (*n* = 289,576)	Yes (*n*= 1,458,557)	
Gender, *n* (%)				0.064
Male	861,301 (49.27%)	143,128 (49.43%)	718,173 (49.24%)	
Female	886,832 (50.73%)	146,448 (50.57%)	740,384 (50.76%)	
Age, mean (SD), years	53.32 (16.87)	45.00 (16.89)	54.97 (16.37)	<0.001 *
				<0.001
20–29 years, *n* (%)	21,3267 (12.20%)	54,748 (18.91%)	158,519 (10.87%)	
30–39 years, *n* (%)	191,879 (10.98%)	72,852 (25.16%)	119,027 (8.16%)	
40–49 years, *n* (%)	231,285 (13.23%)	63,163 (21.81%)	168,122 (11.53%)	
50–59 years, *n* (%)	430,101 (24.60%)	42,637 (14.72%)	387,464 (26.56%)	
60–69 years, *n* (%)	398,344 (22.79%)	28,757 (9.93%)	369,587 (25.34%)	
70–79 years, *n* (%)	190,087 (10.87%)	13,351 (4.61%)	176,736 (12.12%)	
≥80 years, *n* (%)	93,170 (5.33%)	14,068 (4.86%)	79,102 (5.42%)	
Insurance level, *n* (%)				<0.001
Low	449,717 (25.73%)	85,471 (29.52%)	364,246 (24.97%)	
Moderate	492,107 (28.15%)	91,292 (31.53%)	400,815 (27.48%)	
High	806,309 (46.12%)	112,813 (38.96%)	693,496 (47.55%)	
CCI, *n* (%)				<0.001
0	1,226,052 (70.13%)	249,643 (86.21%)	976,409 (66.94%)	
1	274,493 (15.70%)	18,841 (6.51%)	255,652 (17.53%)	
≥2	247,588 (14.16%)	21,092 (7.28%)	226,496 (15.53%)	
Comorbidity, *n* (%)				
DM	249,273 (14.26%)	16,143 (5.57%)	233,130 (15.98%)	<0.001
Hyperlipidemia	507,183 (29.01%)	29,640 (10.24%)	477,543 (32.74%)	<0.001
HTN	459,210 (26.27%)	27,778 (9.59%)	431,432 (29.58%)	<0.001
COPD	59,009 (3.38%)	5414 (1.87%)	53,595 (3.67%)	<0.001
Prior COVID-19 infection, *n* (%)	13,719 (0.78%)	2970 (1.03%)	10,749 (0.74%)	<0.001
1st vaccination product, *n* (%)				
AZD1222	609,023 (41.76%)		609,023 (41.76%)	
BNT162b2	826,953 (56.70%)		826,953 (56.70%)	
mRNA-1273	22,573 (1.55%)		22,573 (1.55%)	
JNJ-78436735	8 (0.00%)		8 (0.00%)	
2nd vaccination product, *n* (%)				
AZD1222	510,261 (34.98%)		510,261 (34.98%)	
BNT162b2	925,692 (63.47%)		925,692 (63.47%)	
mRNA-1273	22,596 (1.55%)		22,596 (1.55%)	
JNJ-78436735	8 (0.00%)		8 (0.00%)	
1st–2nd vaccination product, *n* (%)				
AZD1222–AZD1222	510,253 (34.98%)		510,253 (34.98%)	
AZD1222–BNT162b2	98,762 (6.77%)		98,762 (6.77%)	
AZD1222–mRNA-1273	2 (0.00%)		2 (0.00%)	
AZD1222–JNJ-78436735	6 (0.00%)		6 (0.00%)	
BNT162b2–AZD1222	3 (0.00%)		3 (0.00%)	
BNT162b2–BNT162b2	826,925 (56.69%)		826,925 (56.69%)	
BNT162b2–mRNA-1273	23 (0.00%)		23 (0.00%)	
BNT162b2–JNJ-78436735	2 (0.00%)		2 (0.00%)	
mRNA-1273–BNT162b2	2 (0.00%)		2 (0.00%)	
mRNA-1273–mRNA-1273	22,571 (1.55%)		22,571 (1.55%)	
JNJ-78436735–AZD1222	5 (0.00%)		5 (0.00%)	
JNJ-78436735–BNT162b2	3 (0.00%)		3 (0.00%)	
1st–2nd vaccination type, *n* (%)				
No vaccination	289,576 (16.56%)	289,576 (100%)		
Only mRNA vaccine	849,526 (48.60%)		849,526 (58.24%)	
Only cDNA vaccine	510,253 (29.19%)		510,253 (34.98%)	
Heterologous vaccination	98,778 (5.65%)		98,778 (6.77%)	
Vaccination interval, mean (SD), days	50.88 (23.19)		50.88 (23.19)	

* All values expressed as mean ± standard deviation. *n*, number; CCI, Charson’s comorbidity index; DM, Diabetic mellitus; HTN, Hypertension; COPD, Chronic obstructive pulmonary diseases; AZD-1222, AstraZeneca ChAdOx1-S recombinant vaccine; BNT162b2, Pfizer-BioNTech Comirnaty; mRNA-1273, Moderna Spikevax; JNJ-78436735, Janssen/Johnson and Johnson COVID-19 Vaccine.

**Table 2:** Cumulative incidence rate (cIR) of the broad-spectrum adverse events of special interests (AESIs) following COVID-19 vaccination.

Disease	V	One Week				Two Weeks				One Month				Three Months			
		E	IR	95% CI	*p*	E	cIR	95% CI	*p*	E	cIR	95% CI	*p*	E	cIR	95% CI	*p*
Endometriosis	No	4	0.14	0.00–0.27	0.389	13	0.45	0.20–0.69	0.877	35	1.21	0.81–1.61	0.704	99	3.42	2.75–4.09	0.15
	Yes	34	0.23	0.15–0.31		63	0.43	0.33–0.54		165	1.13	0.96–1.30		584	4	3.68–4.33	
Menstrual disorder	No	82	2.83	2.22–3.44	0.638	161	5.56	4.70–6.42	0.096	346	11.95	10.69–13.21	0.002	1025	35.4	33.23–37.56	<0.001
	Yes	442	3.03	2.75–3.31		935	6.41	6.00–6.82		2081	14.27	13.65–14.88		6481	44.43	43.35–45.51	
Bruise	No	4	0.14	0.00–0.27	<0.001	6	0.21	0.04–0.37	<0.001	11	0.38	0.16–0.60	<0.001	48	1.66	1.19–2.13	<0.001
	Yes	107	0.73	0.59–0.87		181	1.24	1.06–1.42		287	1.97	1.74–2.20		559	3.83	3.51–4.15	
Herpes zoster	No	31	1.07	0.69–1.45	<0.001	66	2.28	1.73–2.83	<0.001	162	5.59	4.73–6.46	<0.001	454	15.68	14.24–17.12	<0.001
	Yes	472	3.24	2.94–3.53		1055	7.23	6.80–7.67		2270	15.56	14.92–16.20		6575	45.08	43.99–46.17	
Alopecia	No	6	0.21	0.04–0.37	0.101	10	0.35	0.13–0.56	0.003	23	0.79	0.47–1.12	<0.001	80	2.76	2.16–3.37	<0.001
	Yes	61	0.42	0.31–0.52		122	0.84	0.69–0.98		266	1.82	1.60–2.04		766	5.25	4.88–5.62	
Warts	No	20	0.69	0.39–0.99	0.279	30	1.04	0.67–1.41	<0.001	78	2.69	2.10–3.29	<0.001	197	6.8	5.85–7.75	<0.001
	Yes	135	0.93	0.77–1.08		303	2.08	1.84–2.31		658	4.51	4.17–4.86		1811	12.42	11.84–12.99	
Visual impairment	No	0	0	0.00–0.00	1.00	0	0	0.00–0.00	0.372	0	0	0.00–0.00	0.024	2	0.07	0.00–0.16	0.008
	Yes	5	0.03	0.00–0.06		9	0.06	0.02–0.10		23	0.16	0.09–0.22		50	0.34	0.25–0.44	
Glaucoma	No	43	1.48	1.04–1.93	<0.001	80	2.76	2.16–3.37	<0.001	199	6.87	5.92–7.83	<0.001	534	18.44	16.88–20.00	<0.001
	Yes	430	2.95	2.67–3.23		901	6.18	5.77–6.58		1892	12.97	12.39–13.56		5749	39.42	38.40–40.43	
Tinnitus	No	11	0.38	0.16–0.60	0.013	26	0.9	0.55–1.24	0.003	54	1.86	1.37–2.36	<0.001	171	5.91	5.02–6.79	<0.001
	Yes	119	0.82	0.67–0.96		237	1.62	1.42–1.83		534	3.66	3.35–3.97		1789	12.27	11.70–12.83	
Inner ear disease	No	43	1.48	1.04–1.93	<0.001	71	2.45	1.88–3.02	<0.001	152	5.25	4.41–6.08	<0.001	466	16.09	14.63–17.55	<0.001
	Yes	553	3.79	3.48–4.11		1094	7.5	7.06–7.94		2381	16.32	15.67–16.98		6870	47.1	45.99–48.21	
Middle ear disease	No	14	0.48	0.23–0.74	<0.001	42	1.45	1.01–1.89	<0.001	93	3.21	2.56–3.86	<0.001	290	10.01	8.86–11.17	<0.001
	Yes	218	1.49	1.30–1.69		468	3.21	2.92–3.50		1058	7.25	6.82–7.69		3343	22.92	22.14–23.70	
Other ear disease	No	43	1.48	1.04–1.93	<0.001	86	2.97	2.34–3.60	<0.001	202	6.98	6.01–7.94	<0.001	607	20.96	19.30–22.63	<0.001
	Yes	550	3.77	3.46–4.09		1112	7.62	7.18–8.07		2441	16.74	16.07–17.40		7552	51.78	50.61–52.94	
Periodontal disease	No	5	0.17	0.02–0.32	1.00	6	0.21	0.04–0.37	0.044	14	0.48	0.23–0.74	0.001	31	1.07	0.69–1.45	<0.001
	Yes	30	0.21	0.13–0.28		70	0.48	0.37–0.59		160	1.1	0.93–1.27		488	3.35	3.05–3.64	

Cumulative incidence rates were calculated as a rate per 10,000 individuals. For the vaccination, the total number was 289,576 non-vaccinated subjects and 1,458,557 vaccinated subjects, respectively V, vaccination; *n*, number; IR, incidence rate; CI, confidence interval; cIR, cumulative incidence rate.

## Data Availability

The data that support the findings of this study are available from the National Health Insurance Service in South Korea, but restrictions apply to the availability of these data, which were used under license for the current study and so are not publicly available. Data are, however, available from the authors upon reasonable request and with permission of the National Health Insurance Service, South Korea.

## Supplementary Materials

The following supporting information can be downloaded at: https://www.mdpi.com/article/10.3390/jcm14051767/s1. Table S1: The cumulative incidence rates of non-fatal immune-related adverse events stratified by gender; Table S2: The cumulative incidence rates of non-fatal immune-related adverse events stratified by vaccine type; Table S3: The risk of non-serious adverse events by gender.

## References

[1] Gupta A., Madhavan M.V., Sehgal K., Nair N., Mahajan S., Sehrawat T.S., Bikdeli B., Ahluwalia N., Ausiello J.C., Wan E.Y. (2020). Extrapulmonary manifestations of COVID-19. Nat. Med..

[2] Feikin D.R., Higdon M.M., Abu-Raddad L.J., Andrews N., Araos R., Goldberg Y., Groome M.J., Huppert A., O’Brien K.L., Smith P.G. (2022). Duration of effectiveness of vaccines against SARS-CoV-2 infection and COVID-19 disease: Results of a systematic review and meta-regression. Lancet.

[3] Voysey M., Clemens S.A.C., Madhi S.A., Weckx L.Y., Folegatti P.M., Aley P.K., Angus B., Baillie V.L., Barnabas S.L., Bhorat Q.E. (2021). Safety and efficacy of the ChAdOx1 nCoV-19 vaccine (AZD1222) against SARS-CoV-2: An interim analysis of four randomised controlled trials in Brazil, South Africa, and the UK. Lancet.

[4] Baden L.R., El Sahly H.M., Essink B., Kotloff K., Frey S., Novak R., Diemert D., Spector S.A., Rouphael N., Creech C.B. (2021). Efficacy and Safety of the mRNA-1273 SARS-CoV-2 Vaccine. N. Engl. J. Med..

[5] Thomas S.J., Moreira E.D., Kitchin N., Absalon J., Gurtman A., Lockhart S., Perez J.L., Pérez Marc G., Polack F.P., Zerbini C. (2021). Safety and Efficacy of the BNT162b2 mRNA Covid-19 Vaccine through 6 Months. N. Engl. J. Med..

[6] Patone M., Handunnetthi L., Saatci D., Pan J., Katikireddi S.V., Razvi S., Hunt D., Mei X.W., Dixon S., Zaccardi F. (2021). Neurological complications after first dose of COVID-19 vaccines and SARS-CoV-2 infection. Nat. Med..

[7] Wang W., Wang C.Y., Wang S.I., Wei J.C. (2022). Long-term cardiovascular outcomes in COVID-19 survivors among non-vaccinated population: A retrospective cohort study from the TriNetX US collaborative networks. EClinicalMedicine.

[8] Cho J.Y., Kim K.H., Lee N., Cho S.H., Kim S.Y., Kim E.K., Park J.H., Choi E.Y., Choi J.O., Park H. (2023). COVID-19 vaccination-related myocarditis: A Korean nationwide study. Eur. Heart J..

[9] Jung J. (2021). Preparing for the Coronavirus Disease (COVID-19) Vaccination: Evidence, Plans, and Implications. J. Korean Med. Sci..

[10] Lee D.S., Kim J.W., Lee K.L., Jung Y.J., Kang H.W. (2022). Adverse events following COVID-19 vaccination in South Korea between February 28 and August 21, 2021: A nationwide observational study. Int. J. Infect. Dis..

[11] Sharifian-Dorche M., Bahmanyar M., Sharifian-Dorche A., Mohammadi P., Nomovi M., Mowla A. (2021). Vaccine-induced immune thrombotic thrombocytopenia and cerebral venous sinus thrombosis post COVID-19 vaccination; a systematic review. J. Neurol. Sci..

[12] Ewer K.J., Barrett J.R., Belij-Rammerstorfer S., Sharpe H., Makinson R., Morter R., Flaxman A., Wright D., Bellamy D., Bittaye M. (2021). T cell and antibody responses induced by a single dose of ChAdOx1 nCoV-19 (AZD1222) vaccine in a phase 1/2 clinical trial. Nat. Med..

[13] Li C., Lee A., Grigoryan L., Arunachalam P.S., Scott M.K.D., Trisal M., Wimmers F., Sanyal M., Weidenbacher P.A., Feng Y. (2022). Mechanisms of innate and adaptive immunity to the Pfizer-BioNTech BNT162b2 vaccine. Nat. Immunol..

[14] Singh R.B., Parmar U.P.S., Kahale F., Agarwal A., Tsui E. (2023). Vaccine-Associated Uveitis after COVID-19 Vaccination: Vaccine Adverse Event Reporting System Database Analysis. Ophthalmology.

[15] Wichova H., Miller M.E., Derebery M.J. (2021). Otologic Manifestations After COVID-19 Vaccination: The House Ear Clinic Experience. Otol. Neurotol..

[16] Fazlollahi A., Zahmatyar M., Noori M., Nejadghaderi S.A., Sullman M.J.M., Shekarriz-Foumani R., Kolahi A.A., Singh K., Safiri S. (2022). Cardiac complications following mRNA COVID-19 vaccines: A systematic review of case reports and case series. Rev. Med. Virol..

[17] Genco L., Cantelli M., Noto M., Battista T., Patrì A., Fabbrocini G., Vastarella M. (2023). Alopecia Areata after COVID-19 Vaccines. Ski. Appendage Disord..

[18] Cazzato G., Romita P., Foti C., Lobreglio D., Trilli I., Colagrande A., Ingravallo G., Resta L. (2022). Development of Flat Warts on the Cheeks after BioNTech-Pfizer BNT162b2 Vaccine: Is There a Correlation?. Vaccines.

[19] Toljan K., Amin M., Kunchok A., Ontaneda D. (2022). New diagnosis of multiple sclerosis in the setting of mRNA COVID-19 vaccine exposure. J. Neuroimmunol..

[20] Gallo G., Mastorino L., Tonella L., Ribero S., Quaglino P. (2022). Alopecia areata after COVID-19 vaccination. Clin. Exp. Vaccine Res..

[21] von Elm E., Altman D.G., Egger M., Pocock S.J., Gøtzsche P.C., Vandenbroucke J.P. (2007). The Strengthening the Reporting of Observational Studies in Epidemiology (STROBE) statement: Guidelines for reporting observational studies. Lancet.

[22] Sundararajan V., Henderson T., Perry C., Muggivan A., Quan H., Ghali W.A. (2004). New ICD-10 version of the Charlson comorbidity index predicted in-hospital mortality. J. Clin. Epidemiol..

[23] Seyed Hosseini E., Riahi Kashani N., Nikzad H., Azadbakht J., Hassani Bafrani H., Haddad Kashani H. (2020). The novel coronavirus Disease-2019 (COVID-19): Mechanism of action, detection and recent therapeutic strategies. Virology.

[24] Lee H.K., Go J., Sung H., Kim S.W., Walter M., Knabl L., Furth P.A., Hennighausen L., Huh J.W. (2022). Heterologous ChAdOx1-BNT162b2 vaccination in Korean cohort induces robust immune and antibody responses that includes Omicron. iScience.

[25] Yonker L.M., Swank Z., Bartsch Y.C., Burns M.D., Kane A., Boribong B.P., Davis J.P., Loiselle M., Novak T., Senussi Y. (2023). Circulating Spike Protein Detected in Post-COVID-19 mRNA Vaccine Myocarditis. Circulation.

[26] Ndeupen S., Qin Z., Jacobsen S., Bouteau A., Estanbouli H., Igyártó B.Z. (2021). The mRNA-LNP platform’s lipid nanoparticle component used in preclinical vaccine studies is highly inflammatory. iScience.

[27] Blix K., Laake I., Juvet L., Robertson A.H., Caspersen I.H., Mjaaland S., Skodvin S.N., Magnus P., Feiring B., Trogstad L. (2023). Unexpected vaginal bleeding and COVID-19 vaccination in nonmenstruating women. Sci. Adv..

[28] Lee K.M.N., Junkins E.J., Luo C., Fatima U.A., Cox M.L., Clancy K.B.H. (2022). Investigating trends in those who experience menstrual bleeding changes after SARS-CoV-2 vaccination. Sci. Adv..

[29] Ciorba A., Corazzi V., Bianchini C., Aimoni C., Pelucchi S., Skarżyński P.H., Hatzopoulos S. (2018). Autoimmune inner ear disease (AIED): A diagnostic challenge. Int. J. Immunopathol. Pharmacol..

[30] Farland L.V., Khan S.M., Shilen A., Heslin K.M., Ishimwe P., Allen A.M., Herbst-Kralovetz M.M., Mahnert N.D., Pogreba-Brown K., Ernst K.C. (2023). COVID-19 vaccination and changes in the menstrual cycle among vaccinated persons. Fertil. Steril..

[31] Su Y.W., Yeh S.J., Chen M.J. (2023). New-onset Glaucoma Following Moderna COVID-19 Vaccination. J. Curr. Glaucoma Pract..

[32] Mulroney T.E., Pöyry T., Yam-Puc J.C., Rust M., Harvey R.F., Kalmar L., Horner E., Booth L., Ferreira A.P., Stoneley M. (2024). N(1)-methylpseudouridylation of mRNA causes +1 ribosomal frameshifting. Nature.

[33] Rahimi Mansour F., Keyvanfar A., Najafiarab H., Rajaei Firouzabadi S., Sefidgar S., Hooshmand Chayijan S., Tarom M., Fadaei M., Farzaneh F., Karimzadeh Bardeei L. (2023). Menstrual disturbances following COVID-19 vaccination: A probable puzzle about the role of endocrine and immune pathways. J. Reprod. Immunol..

[34] Mulroney T.E., Pöyry T., Yam-Puc J.C., Rust M., Harvey R.F., Kalmar L., Horner E., Booth L., Ferreira A.P., Stoneley M. (2021). The Looming Effects of Estrogen in Covid-19: A Rocky Rollout. Front. Nutr..

[35] Kim H.J., Kim M.H., Park S.J., Choi M.G., Chun E.M. (2024). Autoimmune adverse event following COVID-19 vaccination in Seoul, South Korea. J. Allergy Clin. Immunol..

