# Supplementary material for: Broad-Spectrum Adverse Events of Special Interests Based on Immune Response Following COVID-19 Vaccination: A Large-Scale Population-Based Cohort Study
Source: J Clin Med. 2025 Mar 6;14(5):1767. doi: 10.3390/jcm14051767 (PMC11900331; doi:10.3390/jcm14051767)
Supplement: Supplementary file 1 [file jcm-14-01767-s001.zip › Table S1_IRAE_final.pdf]

Table S1. The cumulative incidence rates of non-fatal immune-related adverse events stratified by gender

Disease	Gender	Vaccination	Total	One week				Two weeks				One month				Three months			
			number	event	IR	95% CI	*P*	event	IR	95% CI	*P*	event	IR	95% CI	*P*	event	IR	95% CI	*P*
Endometriosis	Male	No	143128																
		Yes	718173																
	Female	No	146448	4	0.27	0.01-0.54	0.389	13	0.89	0.41-1.37	0.877	35	2.39	1.60-3.18	0.703	99	6.76	5.43-8.09	0.164
		Yes	740384	34	0.46	0.30-0.61		63	0.85	0.64-1.06		165	2.23	1.89-2.57		584	7.89	7.25-8.53	
Menstrual disorder	Male	No	143128																
		Yes	718173																
	Female	No	146448	82	5.6	4.39-6.81	0.638	161	10.99	9.30-12.69	0.112	346	23.63	21.14-26.11	0.003	1025	69.99	65.72-74.26	<0.001
		Yes	740384	442	5.97	5.41-6.53		935	12.63	11.82-13.44		2081	28.11	26.90-29.31		6481	87.54	85.41-89.66	
Bruise	Male	No	143128	1	0.07	0.00-0.21	0.007	2	0.14	0.00-0.33	0.001	3	0.21	0.00-0.45	<0.001	17	1.19	0.62-1.75	<0.001
		Yes	718173	42	0.58	0.41-0.76		65	0.91	0.69-1.13		108	1.5	1.22-1.79		218	3.04	2.63-3.44	
	Female	No	146448	3	0.2	0.00-0.44	0.005	4	0.27	0.01-0.54	<0.001	8	0.55	0.17-0.92	<0.001	31	2.12	1.37-2.86	<0.001
		Yes	740384	65	0.88	0.66-1.09		116	1.57	1.28-1.85		179	2.42	2.06-2.77		341	4.61	4.12-5.09	
Herpes zoster	Male	No	143128	8	0.56	0.17-0.95	<0.001	21	1.47	0.84-2.09	<0.001	59	4.12	3.07-5.17	<0.001	179	12.51	10.68-14.34	<0.001
		Yes	718173	188	2.62	2.24-2.99		411	5.72	5.17-6.28		907	12.63	11.81-13.45		2641	36.77	35.37-38.17	
	Female	No	146448	23	1.57	0.93-2.21	<0.001	45	3.07	2.18-3.97	<0.001	103	7.03	5.68-8.39	<0.001	275	18.78	16.56-21.00	<0.001
		Yes	740384	284	3.84	3.39-4.28		644	8.7	8.03-9.37		1363	18.41	17.43-19.39		3934	53.13	51.48-54.79	
Alopecia	Male	No	143128	2	0.14	0.00-0.33	0.053	5	0.35	0.04-0.66	0.047	11	0.77	0.31-1.22	0.001	38	2.65	1.81-3.50	<0.001
		Yes	718173	38	0.53	0.36-0.70		62	0.86	0.65-1.08		143	1.99	1.66-2.32		373	5.19	4.67-5.72	
	Female	No	146448	4	0.27	0.01-0.54	1	5	0.34	0.04-0.64	0.064	12	0.82	0.36-1.28	0.015	42	2.87	2.00-3.74	<0.001
		Yes	740384	23	0.31	0.18-0.44		60	0.81	0.61-1.02		123	1.66	1.37-1.95		393	5.31	4.78-5.83	
Warts	Male	No	143128	9	0.63	0.22-1.04	0.286	14	0.98	0.47-1.49	0.003	40	2.79	1.93-3.66	<0.001	103	7.2	5.81-8.59	<0.001
		Yes	718173	68	0.95	0.72-1.17		156	2.17	1.83-2.51		349	4.86	4.35-5.37		983	13.69	12.83-14.54	
	Female	No	146448	11	0.75	0.31-1.19	0.649	16	1.09	0.56-1.63	0.02	38	2.59	1.77-3.42	0.005	94	6.42	5.12-7.72	<0.001
		Yes	740384	67	0.9	0.69-1.12		147	1.99	1.66-2.31		309	4.17	3.71-4.64		828	11.18	10.42-11.94	
Visual impairment	Male	No	143128	0	0	0.00-0.00	1	0	0	0.00-0.00	0.598	0	0	0.00-0.00	0.154	0	0	0.00-0.00	0.006
		Yes	718173	3	0.04	0.00-0.09		6	0.08	0.02-0.15		15	0.21	0.10-0.31		31	0.43	0.28-0.58	
	Female	No	146448	0	0	0.00-0.00	1	0	0	0.00-0.00	1	0	0	0.00-0.00	0.368	2	0.14	0.00-0.33	0.561
		Yes	740384	2	0.03	0.00-0.06		3	0.04	0.00-0.09		8	0.11	0.03-0.18		19	0.26	0.14-0.37	
Glaucoma	Male	No	143128	11	0.77	0.31-1.22	<0.001	28	1.96	1.23-2.68	<0.001	78	5.45	4.24-6.66	<0.001	216	15.09	13.08-17.10	<0.001
		Yes	718173	198	2.76	2.37-3.14		393	5.47	4.93-6.01		849	11.82	11.03-12.62		2556	35.59	34.21-36.97	
	Female	No	146448	32	2.19	1.43-2.94	0.056	52	3.55	2.59-4.52	<0.001	121	8.26	6.79-9.73	<0.001	318	21.71	19.33-24.10	<0.001
		Yes	740384	232	3.13	2.73-3.54		508	6.86	6.26-7.46		1043	14.09	13.23-14.94		3193	43.13	41.63-44.62	
Tinnitus	Male	No	143128	5	0.35	0.04-0.66	0.197	12	0.84	0.36-1.31	0.081	25	1.75	1.06-2.43	0.001	73	5.1	3.93-6.27	<0.001
		Yes	718173	48	0.67	0.48-0.86		105	1.46	1.18-1.74		238	3.31	2.89-3.73		789	10.99	10.22-11.75	
	Female	No	146448	6	0.41	0.08-0.74	0.044	14	0.96	0.46-1.46	0.025	29	1.98	1.26-2.70	<0.001	98	6.69	5.37-8.02	<0.001
		Yes	740384	71	0.96	0.74-1.18		132	1.78	1.48-2.09		296	4	3.54-4.45		1000	13.51	12.67-14.34	
Inner ear disease	Male	No	143128	13	0.91	0.41-1.40	<0.001	20	1.4	0.78-2.01	<0.001	48	3.35	2.41-4.30	<0.001	147	10.27	8.61-11.93	<0.001
		Yes	718173	171	2.38	2.02-2.74		355	4.94	4.43-5.46		798	11.11	10.34-11.88		2219	30.9	29.61-32.18	
	Female	No	146448	30	2.05	1.32-2.78	<0.001	51	3.48	2.53-4.44	<0.001	104	7.1	5.74-8.47	<0.001	319	21.78	19.39-24.17	<0.001
		Yes	740384	382	5.16	4.64-5.68		739	9.98	9.26-10.70		1583	21.38	20.33-22.43		4651	62.82	61.02-64.62	
Middle ear disease	Male	No	143128	5	0.35	0.04-0.66	<0.001	15	1.05	0.52-1.58	<0.001	39	2.72	1.87-3.58	<0.001	126	8.8	7.27-10.34	<0.001
		Yes	718173	102	1.42	1.14-1.70		217	3.02	2.62-3.42		495	6.89	6.29-7.50		1495	20.82	19.76-21.87	
	Female	No	146448	9	0.61	0.21-1.02	0.004	27	1.84	1.15-2.54	0.002	54	3.69	2.70-4.67	<0.001	164	11.2	9.49-12.91	<0.001
		Yes	740384	116	1.57	1.28-1.85		251	3.39	2.97-3.81		563	7.6	6.98-8.23		1848	24.96	23.82-26.10	
Other ear disease	Male	No	143128	19	1.33	0.73-1.92	<0.001	32	2.24	1.46-3.01	<0.001	84	5.87	4.61-7.12	<0.001	267	18.65	16.42-20.89	<0.001
		Yes	718173	260	3.62	3.18-4.06		526	7.32	6.70-7.95		1134	15.79	14.87-16.71		3460	48.18	46.58-49.78	
	Female	No	146448	24	1.64	0.98-2.29	<0.001	54	3.69	2.70-4.67	<0.001	118	8.06	6.60-9.51	<0.001	340	23.22	20.75-25.68	<0.001
		Yes	740384	290	3.92	3.47-4.37		586	7.91	7.27-8.56		1307	17.65	16.70-18.61		4092	55.27	53.58-56.96	
Periodontal disease	Male	No	143128	0	0	0.00-0.00	0.037	0	0	0.00-0.00	0.003	5	0.35	0.04-0.66	0.01	11	0.77	0.31-1.22	<0.001
		Yes	718173	21	0.29	0.17-0.42		36	0.5	0.34-0.67		75	1.04	0.81-1.28		247	3.44	3.01-3.87	
	Female	No	146448	5	0.34	0.04-0.64	0.067	6	0.41	0.08-0.74	1	9	0.61	0.21-1.02	0.071	20	1.37	0.77-1.96	<0.001
		Yes	740384	9	0.12	0.04-0.20		34	0.46	0.30-0.61		85	1.15	0.90-1.39		241	3.26	2.84-3.67	
