# Supplementary material for: Broad-Spectrum Adverse Events of Special Interests Based on Immune Response Following COVID-19 Vaccination: A Large-Scale Population-Based Cohort Study
Source: J Clin Med. 2025 Mar 6;14(5):1767. doi: 10.3390/jcm14051767 (PMC11900331; doi:10.3390/jcm14051767)
Supplement: Supplementary file 1 [file jcm-14-01767-s001.zip › Table S2_IRAE_final.pdf]

Table S2. The cumulative incidence rates of non-fatal immune-related adverse events stratified by vaccine type

Disease	Vaccination	Total number	One week				Two weeks				One month				Three months			
			event	IR	95% CI	*P*	event	IR	95% CI	*P*	event	IR	95% CI	*P*	event	IR	95% CI	*P*
Endometriosis	No	289576	4	0.14	0.00-0.27	0.264	13	0.45	0.20-0.69	0.213	35	1.21	0.81-1.61	0.002	99	3.42	2.75-4.09	<0.001
	only mRNA vaccine	849526	22	0.26	0.15-0.37		41	0.48	0.33-0.63		109	1.28	1.04-1.52		401	4.72	4.26-5.18	
	only cDNA vaccine	510253	8	0.16	0.05-0.27		15	0.29	0.15-0.44		37	0.73	0.49-0.96		97	1.9	1.52-2.28	
	Cross	98778	4	0.4	0.01-0.80		7	0.71	0.18-1.23		19	1.92	1.06-2.79		86	8.71	6.87-10.55	
Menstrual disorder	No	289576	82	2.83	2.22-3.44	<0.001	161	5.56	4.70-6.42	<0.001	346	11.95	10.69-13.21	<0.001	1025	35.4	33.23-37.56	<0.001
	only mRNA vaccine	849526	334	3.93	3.51-4.35		685	8.06	7.46-8.67		1533	18.05	17.14-18.95		4891	57.57	55.96-59.18	
	only cDNA vaccine	510253	63	1.23	0.93-1.54		138	2.7	2.25-3.16		281	5.51	4.86-6.15		810	15.87	14.78-16.97	
	Cross	98778	45	4.56	3.22-5.89		112	11.34	9.24-13.44		267	27.03	23.79-30.27		780	78.96	73.45-84.48	
Bruise	No	289576	4	0.14	0.00-0.27	<0.001	6	0.21	0.04-0.37	<0.001	11	0.38	0.16-0.60	<0.001	48	1.66	1.19-2.13	<0.001
	only mRNA vaccine	849526	56	0.66	0.49-0.83		86	1.01	0.80-1.23		142	1.67	1.40-1.95		296	3.48	3.09-3.88	
	only cDNA vaccine	510253	47	0.92	0.66-1.18		87	1.71	1.35-2.06		131	2.57	2.13-3.01		231	4.53	3.94-5.11	
	Cross	98778	4	0.4	0.01-0.80		8	0.81	0.25-1.37		14	1.42	0.67-2.16		32	3.24	2.12-4.36	
Herpes zoster	No	289576	31	1.07	0.69-1.45	<0.001	66	2.28	1.73-2.83	<0.001	162	5.59	4.73-6.46	<0.001	454	15.68	14.24-17.12	<0.001
	only mRNA vaccine	849526	237	2.79	2.43-3.14		535	6.3	5.76-6.83		1136	13.37	12.60-14.15		3365	39.61	38.27-40.95	
	only cDNA vaccine	510253	215	4.21	3.65-4.78		463	9.07	8.25-9.90		987	19.34	18.14-20.55		2760	54.09	52.08-56.10	
	Cross	98778	20	2.02	1.14-2.91		57	5.77	4.27-7.27		147	14.88	12.48-17.29		450	45.56	41.36-49.76	
Alopecia	No	289576	6	0.21	0.04-0.37	0.024	10	0.35	0.13-0.56	0.001	23	0.79	0.47-1.12	<0.001	80	2.76	2.16-3.37	<0.001
	only mRNA vaccine	849526	41	0.48	0.33-0.63		82	0.97	0.76-1.17		175	2.06	1.75-2.37		482	5.67	5.17-6.18	
	only cDNA vaccine	510253	13	0.25	0.12-0.39		28	0.55	0.35-0.75		63	1.23	0.93-1.54		195	3.82	3.29-4.36	
	Cross	98778	7	0.71	0.18-1.23		12	1.21	0.53-1.90		28	2.83	1.78-3.88		89	9.01	7.14-10.88	
Warts	No	289576	20	0.69	0.39-0.99	0.052	30	1.04	0.67-1.41	<0.001	78	2.69	2.10-3.29	<0.001	197	6.8	5.85-7.75	<0.001
	only mRNA vaccine	849526	89	1.05	0.83-1.27		197	2.32	2.00-2.64		433	5.1	4.62-5.58		1157	13.62	12.84-14.40	
	only cDNA vaccine	510253	34	0.67	0.44-0.89		79	1.55	1.21-1.89		173	3.39	2.89-3.90		504	9.88	9.02-10.74	
	Cross	98778	12	1.21	0.53-1.90		27	2.73	1.70-3.76		52	5.26	3.83-6.69		150	15.19	12.76-17.61	
Visual impairment	No	289576	0	0	0.00-0.00	0.421	0	0	0.00-0.00	0.08	0	0	0.00-0.00	0.012	2	0.07	0.00-0.16	<0.001
	only mRNA vaccine	849526	2	0.02	0.00-0.06		3	0.04	0.00-0.08		8	0.09	0.03-0.16		18	0.21	0.11-0.31	
	only cDNA vaccine	510253	3	0.06	0.00-0.13		6	0.12	0.02-0.21		13	0.25	0.12-0.39		30	0.59	0.38-0.80	
	Cross	98778	0	0	0.00-0.00		0	0	0.00-0.00		2	0.2	0.00-0.48		2	0.2	0.00-0.48	
Glaucoma	No	289576	43	1.48	1.04-1.93	<0.001	80	2.76	2.16-3.37	<0.001	199	6.87	5.92-7.83	<0.001	534	18.44	16.88-20.00	<0.001
	only mRNA vaccine	849526	218	2.57	2.23-2.91		449	5.29	4.80-5.77		966	11.37	10.65-12.09		3034	35.71	34.45-36.98	
	only cDNA vaccine	510253	186	3.65	3.12-4.17		389	7.62	6.87-8.38		798	15.64	14.56-16.72		2367	46.39	44.52-48.25	
	Cross	98778	26	2.63	1.62-3.64		63	6.38	4.80-7.95		128	12.96	10.71-15.20		348	35.23	31.54-38.93	
Tinnitus	No	289576	11	0.38	0.16-0.60	0.019	26	0.9	0.55-1.24	0.002	54	1.86	1.37-2.36	<0.001	171	5.91	5.02-6.79	<0.001
	only mRNA vaccine	849526	72	0.85	0.65-1.04		138	1.62	1.35-1.90		316	3.72	3.31-4.13		991	11.67	10.94-12.39	
	only cDNA vaccine	510253	44	0.86	0.61-1.12		92	1.8	1.43-2.17		195	3.82	3.29-4.36		708	13.88	12.85-14.90	
	Cross	98778	3	0.3	0.00-0.65		7	0.71	0.18-1.23		23	2.33	1.38-3.28		90	9.11	7.23-10.99	
Inner ear disease	No	289576	43	1.48	1.04-1.93	<0.001	71	2.45	1.88-3.02	<0.001	152	5.25	4.41-6.08	<0.001	466	16.09	14.63-17.55	<0.001
	only mRNA vaccine	849526	297	3.5	3.10-3.89		582	6.85	6.29-7.41		1285	15.13	14.30-15.95		3735	43.97	42.56-45.37	
	only cDNA vaccine	510253	222	4.35	3.78-4.92		456	8.94	8.12-9.76		977	19.15	17.95-20.35		2793	54.74	52.71-56.76	
	Cross	98778	34	3.44	2.29-4.60		56	5.67	4.18-7.15		119	12.05	9.88-14.21		342	34.62	30.96-38.29	
Middle ear disease	No	289576	14	0.48	0.23-0.74	<0.001	42	1.45	1.01-1.89	<0.001	93	3.21	2.56-3.86	<0.001	290	10.01	8.86-11.17	<0.001
	only mRNA vaccine	849526	125	1.47	1.21-1.73		284	3.34	2.95-3.73		623	7.33	6.76-7.91		1973	23.22	22.20-24.25	
	only cDNA vaccine	510253	82	1.61	1.26-1.95		158	3.1	2.61-3.58		372	7.29	6.55-8.03		1157	22.68	21.37-23.98	
	Cross	98778	11	1.11	0.46-1.77		26	2.63	1.62-3.64		63	6.38	4.80-7.95		213	21.56	18.67-24.46	
Other ear disease	No	289576	43	1.48	1.04-1.93	<0.001	86	2.97	2.34-3.60	<0.001	202	6.98	6.01-7.94	<0.001	607	20.96	19.30-22.63	<0.001
	only mRNA vaccine	849526	323	3.8	3.39-4.22		655	7.71	7.12-8.30		1448	17.04	16.17-17.92		4309	50.72	49.21-52.23	
	only cDNA vaccine	510253	200	3.92	3.38-4.46		408	8	7.22-8.77		886	17.36	16.22-18.51		2881	56.46	54.41-58.52	
	Cross	98778	27	2.73	1.70-3.76		49	4.96	3.57-6.35		107	10.83	8.78-12.88		362	36.65	32.88-40.42	
	No	289576	5	0.17	0.02-0.32	0.081	6	0.21	0.04-0.37	<0.001	14	0.48	0.23-0.74	<0.001	31	1.07	0.69-1.45	<0.001

Periodontal disease	only mRNA vaccine	849526	11	0.13	0.05-0.21		26	0.31	0.19-0.42		65	0.77	0.58-0.95		226	2.66	2.31-3.01	
	only cDNA vaccine	510253	17	0.33	0.17-0.49		37	0.73	0.49-0.96		82	1.61	1.26-1.95		228	4.47	3.89-5.05	
	Cross	98778	2	0.2	0.00-0.48		7	0.71	0.18-1.23		13	1.32	0.60-2.03		34	3.44	2.29-4.60	
