# Supplementary material for: Broad-Spectrum Adverse Events of Special Interests Based on Immune Response Following COVID-19 Vaccination: A Large-Scale Population-Based Cohort Study
Source: J Clin Med. 2025 Mar 6;14(5):1767. doi: 10.3390/jcm14051767 (PMC11900331; doi:10.3390/jcm14051767)
Supplement: Supplementary file 1 [file jcm-14-01767-s001.zip › Table S3_COVIDirAE_final.pdf]

Table S3 The risk of non-serious adverse events by genders

Disease	Multiple logistic regression model												Cox proportional model		
	One week			Two weeks			One month			Three months					
	OR	95% CI	*P*	OR	95% CI	*P*	OR	95% CI	*P*	OR	95% CI	*P*	HR	95% CI	*P*
Bruise	1.52	1.03-2.23	0.035	1.73	1.28-2.34	< 0.001	1.62	1.28-2.06	< 0.001	1.53	1.30-1.80	< 0.001	1.53	1.23-1.80	< 0.001
Herpes zoster	1.49	1.24-1.78	< 0.001	1.51	1.34-1.71	< 0.001	1.44	1.33-1.57	< 0.001	1.42	1.35-1.49	< 0.001	1.42	1.35-1.49	< 0.001
Alopecia	0.67	0.41-1.09	0.107	0.93	0.66-1.31	0.659	0.83	0.66-1.05	0.120	1.01	0.88-1.16	0.852	1.01	0.86-1.16	0.847
Warts	0.99	0.72-1.35	0.927	0.97	0.78-1.20	0.779	0.88	0.76-1.02	0.089	0.84	0.77-0.92	< 0.001	0.84	0.77-0.92	< 0.001
Visual impairment	0.57	0.09-3.51	0.544	0.46	0.11-1.87	0.278	0.49	0.21-1.17	0.110	0.64	0.37-1.12	0.116	0.66	0.38-1.15	0.139
Glaucoma	1.22	1.01-1.46	0.036	1.29	1.13-1.46	< 0.001	1.21	1.11-1.32	< 0.001	1.23	1.16-1.29	< 0.001	1.23	1.17-1.29	< 0.001
Tinnitus	1.38	0.97-1.96	0.075	1.19	0.93-1.52	0.162	1.18	1.00-1.39	0.048	1.22	1.11-1.33	< 0.001	1.22	1.12-1.33	< 0.001
Inner ear disease	2.14	1.79-2.55	< 0.001	2.01	1.78-2.28	< 0.001	1.91	1.75-2.07	< 0.001	2.00	1.91-2.10	< 0.001	2.00	1.90-2.10	< 0.001
Middle ear disease	1.15	0.88-1.49	0.302	1.18	0.99-1.40	0.072	1.13	1.00-1.27	0.043	1.21	1.13-1.29	< 0.001	1.21	1.13-1.29	< 0.001
Other ear disease	1.05	0.90-1.24	0.525	1.09	0.97-1.22	0.146	1.11	1.03-1.20	0.008	1.13	1.09-1.18	< 0.001	1.13	1.08-1.18	< 0.001
Periodontal disease	0.64	0.32-1.26	0.195	1.09	0.69-1.71	0.721	1.14	0.84-1.53	0.406	0.96	0.81-1.14	0.632	0.96	0.81-1.14	0.627

The values were presented as comparisons of females to males. OR, odd ratio; CI, confidence interval; HR, hazard ratio.
